# Retrospective Analysis of the Epidemiology and Risk Factors for Recurrent Biliary-Source Bloodstream Infections in Oncologic Patients

**DOI:** 10.3390/antibiotics15040342

**Published:** 2026-03-27

**Authors:** Paola Maffezzoli, Ignacio Grafia, Mar Cusó Banús, Aina Gutiérrez-Santos, Alba Fernández, Ana Peris, Laia Llobera, Maria Dolores Quesada, Daniela Buccione, Silvia Corcione, Carolina Tudela, Carme Bracke, Anna Esquerrà, Alba Romero, Gabriela Cerdà, Rosa Benítez, Aina Mateu, Anna Sales, Alex Soriano, Roger Paredes, Pere-Joan Cardona, Francesco Giuseppe De Rosa, María Luisa Pedro-Botet, Pedro Puerta-Alcalde

**Affiliations:** 1 Department of Medical Sciences, Internal Medicine, University of Turin, 10124 Turin, Italy; paolamaffezzoli94@gmail.com (P.M.); francescogiuseppe.derosa@unito.it (F.G.D.R.); 2 Department of Infectious Diseases, Hospital Universitari Germans Trias i Pujol, 08916 Badalona, Spain; igrafia@lluita.org (I.G.); aperis@lluita.org (A.P.); cbracke@lluita.org (C.B.); aromero@lluita.org (A.R.); rbenitez@lluita.org (R.B.); rparedes@lluita.org (R.P.); mlpbotet.germanstrias@gencat.cat (M.L.P.-B.); 3 Department of Medicine, Universitat de Barcelona (UB), 08036 Barcelona, Spain; 4 Department of Internal Medicine, Hospital Universitari Germans Trias i Pujol, 08916 Badalona, Spain; cusomar9@gmail.com (M.C.B.); ainagutierrezsantos@gmail.com (A.G.-S.); 5 Department of Dermatology, Hospital Universitari Germans Trias i Pujol, 08916 Badalona, Spain; ferpialba@gmail.com; 6 Department of Oncology, Hospital Universitari Germans Trias i Pujol, ICO, 08916 Badalona, Spain; laiallobera@iconocologia.net (L.L.); dbuccione@iconcologia.net (D.B.); carol.tudela@gmail.com (C.T.); aesquerram@iconcologia.net (A.E.); gcerda@iconcologia.net (G.C.); amateus@iconcologia.net (A.M.); asalem@iconcologia.net (A.S.); 7 Department of Clinical Microbiology, Hospital Universitari Germans Trias i Pujol, 08916 Badalona, Spain; mdquesada.germanstrias@gencat.cat (M.D.Q.); pcardonai.germanstrias@gencat.cat (P.-J.C.); 8 Department of Medical Sciences, Infectious Diseases, University of Turin, 10126 Turin, Italy; silvia.corcione@unito.it; 9 Division of Geographic Medicine, Tufts University School of Medicine, Boston, MA 02111, USA; 10 Infectious Diseases Department, Hospital Clínic-IDIBAPS, 08036 Barcelona, Spain; asoriano@clinic.cat; 11 CIBERINF Ciber in Infectious Diseases, ISCIII, Madrid 28029, Spain; 12 Fundació Lluita Contra les Infeccions, 08916 Badalona, Spain; 13 Department of Medicine, University of Vic (UVic), 08500 Vic, Spain

**Keywords:** cholangitis, bacteremia, biliary-source bloodstream infection, mortality, empirical treatment

## Abstract

**Background**: We aimed to describe the clinical and microbiological characteristics of biliary-source bloodstream infections (bBSIs) in patients with malignancies and identify risk factors for recurrence. **Methods**: All bBSI episodes in patients with active solid tumors during 2021–2025 were retrospectively reviewed. Independent risk factors for recurrent bBSI and mortality were identified. A previously published recurrence risk score was externally validated. **Results**: Overall, 136 patients experienced 199 bBSI episodes. Pancreatic (36.7%) and biliary tract (33.2%) were the most common cancers, and 60.8% had metastatic disease. The main pathogens were *Escherichia coli* (43.2%), *Klebsiella pneumoniae* (24.1%), and *Enterococcus faecium* (19.1%), and multidrug-resistant organisms accounted for 19.1%. Inappropriate empirical antibiotic treatment (IEAT) occurred in 37.2% and was independently associated with increased 30-day mortality, together with metastatic disease and septic shock. Thirty-day mortality was 24.6%. Recurrent bBSI occurred in 35.7% and was independently associated with biliary tract cancer, previous multidrug-resistant isolation, and prior hospitalization for suspected biliary infection. The externally validated recurrence score showed excellent discrimination (AUC 0.815). **Conclusions**: bBSI in oncology patients is associated with high rates of MDR pathogens, IEAT, recurrence, and mortality. A simple clinical score may identify patients at high risk of recurrence and guide preventive strategies.

## 1. Introduction

Bloodstream infections originating from the biliary tract (bBSIs) are a significant complication in oncology patients, typically arising from primary tumors, metastatic involvement, or biliary obstruction due to adenopathy [1,2,3]. In addition to bacteremia-related mortality, bBSIs are associated with substantial morbidity, including hepatic abscesses, the need for biliary decompression procedures, and delays or discontinuation of chemotherapy [4].

The rising global prevalence of multidrug-resistant (MDR) Gram-negative bacilli (GNB) poses a major challenge for patients with malignancies, in whom recurrent healthcare exposures and repeated courses of broad-spectrum antibiotics markedly increase selective pressure [5,6,7,8]. As a result, selecting appropriate empirical antibiotic therapy has become increasingly difficult. This is particularly relevant, as inadequate empirical treatment has been associated with increased mortality and higher healthcare costs [9,10,11].

A particularly important problem in patients with bBSI is the high rate of recurrence. A recent study reported that nearly 30% of oncology patients with bBSI had experienced a previous episode of biliary bacteremia [12]. Independent risk factors for recurrent bBSIs included prior antibiotic exposure, the presence of a biliary prosthesis, prior hospital admission for suspected biliary infection, and an index BSI episode caused by an MDR-GNB. Based on these variables and the corresponding regression coefficients, the authors proposed a simple score to identify patients at increased risk of recurrence after a first episode, assigning 1 point to biliary prosthesis and MDR-GNB infection and 2 points to prior antibiotic exposure and prior admission due to suspected biliary infection. However, external validation of this score was lacking.

We aimed to identify independent risk factors for recurrence in a cohort of oncologic patients with bBSIs and externally validate the previously described prediction score. As a secondary objective, we sought to characterize our cohort by assessing the prevalence of MDR microorganisms, the appropriateness of empirical antibiotic therapy, and the occurrence of secondary complications, as well as identifying risk factors associated with mortality.

## 2. Results

### 2.1. Patient Demographics and Clinical Characteristics

A total of 199 episodes of biliary-source bloodstream infections (bBSIs) were identified in 136 patients with active malignancy. The median patient age was 71 years (IQR 64–76), and 61% were male. Table 1 summarizes the demographic characteristics, major comorbidities, and oncologic features of the cohort. At least one comorbidity was present in 63.3% of patients. The most common underlying malignancies were pancreatic cancer (36.7%), biliary tract cancer (33.2%), and colorectal cancer (10.6%). Metastatic disease was present in 60.8% of patients, and biliary tract involvement was directly related to the primary tumor in 69.3% of cases. Prior biliary manipulation, including ERCP or PTC, occurred in 60.8% of cases, and biliary prosthesis was present in 64.3% of patients.

### 2.2. Microbiological Findings and Patterns of Antimicrobial Resistance

Table 2 summarizes the causative pathogens and their resistance profiles. Most episodes (160, 80.4%) were due to Gram-negative bacilli, with Gram-positive cocci in 56 episodes (28.1%), with 3 cases of candidemia (1.5%), and 41 polymicrobial infections (20.6%). The predominant pathogen was *Escherichia coli* (86 episodes, 43.2%), of which 22.1% were ESBL-producing strains and 2.3% were carbapenem-resistant. *Klebsiella pneumoniae* was the second most frequent pathogen (48 episodes, 24.1%), with 25% ESBL producers and 2.1% carbapenem-resistant isolates. *Enterococcus faecium* accounted for 38 episodes (19.1%). Overall, MDR Gram-negative bacilli caused 19.1% of all bBSI episodes.

### 2.3. Appropriateness of Empirical Antibiotic Therapy and Outcomes

**Figure 1:**
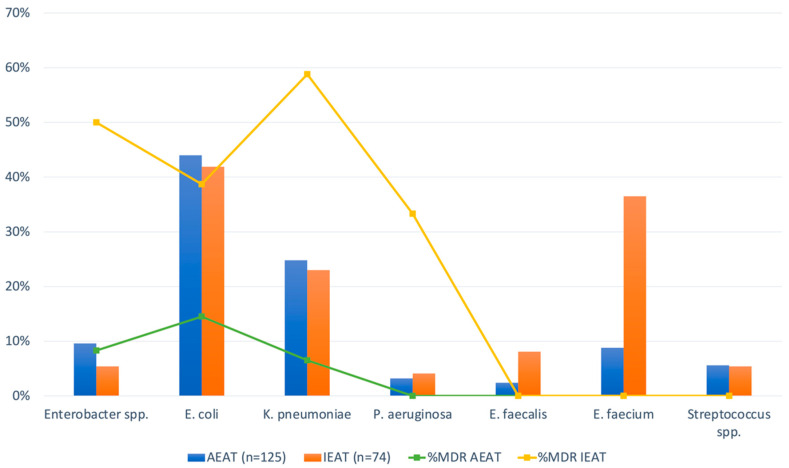
Causative pathogens and multidrug resistance according to appropriateness of empirical antibiotic therapy. Bars represent the percentage of bloodstream infection episodes caused by each microorganism according to appropriateness of empirical therapy. Lines indicate the proportion of multidrug-resistant (MDR) isolates. Abbreviations. AEAT: appropriate empirical antibiotic treatment; IEAT: inappropriate empirical antibiotic treatment; MDR: multidrug-resistant.

Supplementary Tables S1 and S2 summarize the most frequently used empirical and definitive antibiotic treatments, respectively. Table 3 summarizes the main outcomes of the bBSI episodes. Inappropriate empirical antibiotic treatment (IEAT) occurred in 74 cases (37.2%). Figure 1 shows the causative pathogens and multidrug-resistance profiles according to the appropriateness of empirical antibiotic therapy. Secondary hepatic abscesses developed in 19.1% of patients following the bBSI episode. Among patients receiving oncologic therapy, treatment was delayed in 25.3% of cases and permanently discontinued in 52.7% due to the infection. Septic shock occurred in 19.1% of episodes. The overall 30-day mortality rate was 24.6%, with 75.5% of deaths directly attributable to the bBSI.

### 2.4. Risk Factors for Mortality

Table 1, Table 2 and Table 3 present the univariate analysis of risk factors for mortality. In the multivariable model, independent predictors of increased 30-day mortality included the presence of metastatic disease (OR 2.731, 95% CI 1.220–6.114), IEAT (OR 2.182, 95% CI 1.048–4.543), and septic shock (OR 2.872, 95% CI 1.227–6.718). Conversely, receiving definitive treatment with ciprofloxacin (OR 0.064, 95% CI 0.008–0.503) and prior chemotherapy (OR 0.337, 95% CI 0.153–0.741) were independently associated with lower mortality. The predictive model demonstrated moderate discriminatory performance, with an area under the ROC curve of 0.780 (95% CI 0.710–0.849). Bootstrap analysis confirmed the direction of these associations, although some variables (e.g., shock and IEAT) showed borderline significance.

### 2.5. Recurrent Episodes

In our cohort, 35.7% of patients had a previous biliary-source BSI and 41.7% had been hospitalized in the prior year for a suspected biliary infection without documented bacteremia. Overall, 48 patients experienced recurrent episodes and were compared with 88 non-recurrent cases (Table 1, Table 2 and Table 3). In multivariable analysis, three independent risk factors for recurrent bBSIs were identified: biliary tract cancer (OR 3.995, 95% CI 1.269–12.577), prior admission for suspected biliary infection (OR 27.610, 95% CI 9.120–83.588), and previous isolation of an MDR organism within the preceding six months (OR 9.864, 95% CI 1.982–49.081). Bootstrap analysis showed wider confidence intervals, particularly for biliary tract cancer, reflecting residual uncertainty in effect estimates. The predictive model showed excellent discriminatory performance, with an area under the ROC curve of 0.892 (95% CI 0.835–0.948).

To evaluate the applicability of the previously published recurrence score, we applied it to the 136 individual patients in our cohort. The score yielded an AUC of 0.815 (95% CI 0.738–0.892), confirming strong predictive capacity, though slightly lower than that of our multivariable model. Table 4 summarizes the diagnostic performance across different cutoff points, including sensitivities, specificities, and predictive values, allowing optimization of recurrence prediction based on the characteristics of our population.

## 3. Discussion

We conducted a retrospective cohort study including 199 bBSI episodes in 136 patients with active malignancy. The main findings were as follows: (i) Gram-negative bacilli were the predominant causative pathogens, with MDR strains accounting for nearly one-fifth of cases; (ii) oncologic treatment was frequently delayed or permanently discontinued following the bBSI episode; (iii) inappropriate empirical antibiotic therapy (IEAT) was common (37.2%), particularly in episodes caused by MDR pathogens; (iv) the 30-day mortality rate was high (24.6%) and was independently associated with IEAT; (v) recurrent bBSI episodes occurred in more than one-third of patients and were independently associated with biliary tract cancer, prior hospitalization for suspected biliary infection, and prior MDR isolation; and (vi) the previously published recurrence score accurately identified patients at higher risk of recurrence in our cohort.

Similar to other studies of cancer patients with bBSI [2], our cohort consisted of relatively older individuals with a substantial burden of comorbidities, high rates of metastatic disease, and frequent biliary tract involvement related to the underlying malignancy. This profile is comparable to that reported by Grafia et al. [12], in whom the original recurrence score was developed: median age was similar (68 years vs. 71 years in our cohort), primary tumor involvement of the biliary tract was observed in 63% of cases compared with 69% in our study, and approximately 40% of patients had relevant comorbidities.

Consistent with other published cohorts [2,12], bBSIs in our study were mainly caused by GNB, predominantly *E. coli* and *K. pneumoniae*. The prevalence of MDR organisms was high, accounting for nearly one fifth of all episodes, and one quarter of those caused by GNB. In this context, IEAT was unacceptably frequent, occurring in almost 40% of cases, particularly in episodes involving MDR-GNB and *E. faecium*. In the study by Royo-Cebrecos et al. [2], the proportion of MDR-GNB was lower (11.4% of all episodes), and accordingly, the rate of IEAT was also lower (23.1%). In contrast, Grafia et al. [12] reported MDR rates comparable to those observed in our cohort (21.5%), yet IEAT rates remained substantially lower (23.8%). Although empirical antibiotic regimens were not detailed in their cohort, these discrepancies may be largely explained by local prescribing practices in our cohort, including the relatively low use of carbapenems (22.6%) despite nearly a quarter of *E. coli* and *K. pneumoniae* isolates being ESBL-producing, and the infrequent empirical coverage of *E. faecium* (13.1%) despite it being the third most common pathogen overall. Notably, *E. faecium* was the second most frequently isolated pathogen among patients who received IEAT. Most importantly, IEAT was independently associated with higher mortality, highlighting the importance of tailoring empirical therapy to local epidemiology, particularly in severely ill patients. Interestingly, ciprofloxacin as definitive treatment was linked to lower mortality, likely reflecting its use in stable, orally tolerant patients, and possibly benefiting from its anti-biofilm activity and excellent tissue penetration in settings with biliary prostheses and secondary abscesses. However, the relatively low number of events and the width of some confidence intervals warrant cautious interpretation of these findings, which should be explored in future studies.

Factors independently associated with recurrent bBSIs were biliary tract malignancy, prior hospitalization for suspected biliary infection, and prior isolation of a MDR organism, consistent with findings from the Grafia et al. cohort [12]. Biliary tract cancer may induce structural alterations, while biliary prostheses provide surfaces that favor biofilm formation on both ductal walls and prosthetic devices [13]. Prior biliary infection episodes were a common risk factor for recurrence in both cohorts, likely reflecting underlying structural predisposition and persistent biliary colonization. Similarly, both cohorts identified MDR infection or colonization as a significant risk factor. MDR colonization is often a marker of profoundly disrupted microbiota, in which benign species are displaced by pathogenic organisms that are difficult to eradicate [3,4,5,6,7]. The persistence of these pathogens within the biliary tract—either within prosthetic biofilms and/or due to major duodenal papilla dysfunction—facilitates recurrent bBSI episodes [13,14]. However, the magnitude of some effect estimates should be interpreted cautiously given the limited number of outcome events and the resulting uncertainty.

External validation of the previous score showed good performance in our cohort. According to the score, patients with very low values (≤2) had a very low likelihood of recurrence (negative predictive value [NPV] 92.2%), whereas those with scores >4 had a very high likelihood of recurrence (positive predictive value [PPV] 70.6%), while still maintaining a moderate NPV (76.5%). Given the high recurrence rates observed in both our cohort and in previous bBSI studies, we believe that a cutoff >2 (PPV 65.6%, NPV 89.3%) could be used as a simplified bedside tool to guide decisions regarding antibiotic prophylaxis in this high-risk population. The potential benefit of additional strategies, such as use of antibiotics with high anti-biofilm activity [15], or scheduled biliary prosthesis exchange, warrants further evaluation.

The strengths of our study include the inclusion of a large number of biliary bacteremia episodes in oncological patients and the external validation of a previously developed score based on simple clinical variables. However, several limitations should be acknowledged. First, this was a single-center retrospective study, and our results may have been influenced by center-specific factors, including local pathogen distribution, antimicrobial resistance patterns, and institutional empirical prescribing practices. These factors may differ across healthcare settings and should be considered when extrapolating our findings to other settings. Second, the score was originally developed in the same geographic area, which may limit the generalizability of our findings to centers with different epidemiological profiles. Third, no genomic analysis was performed to determine whether recurrent episodes caused by the same pathogen represented relapse with the same strain or reinfection due to a different strain. Additionally, only episodes with microbiologically confirmed bBSIs were included, potentially excluding febrile episodes without documented bacteremia. Future multicenter, prospective studies are needed to confirm these results and to assess whether targeted prophylaxis for high-risk patients identified by the score can reduce recurrence rates and provide clinical benefit. In addition, important clinical variables such as severity scores (e.g., SOFA or Pitt bacteremia score) and timing of source control, including biliary drainage, could not be systematically evaluated due to the retrospective design and incomplete data availability. Finally, the episode-level analysis for mortality did not account for potential within-patient correlation arising from multiple episodes in the same individual, which may have led to underestimation of standard errors.

As a conclusion, in patients with active malignancy, bBSIs are frequently caused by GNB, often involve MDR organisms, and are associated with high rates of inappropriate empirical therapy, recurrence, and mortality. IEAT emerged as the main modifiable factor independently associated with death. Recurrent infections were common and driven by biliary tract malignancy, prior biliary infection, and previous MDR isolation. A simple clinical score reliably identified patients at high risk of recurrence and may help guide preventive strategies in this vulnerable population.

## 4. Materials and Methods

### 4.1. Study Design, Setting and Population

This retrospective study was conducted at Hospital Germans Trias i Pujol, a tertiary-care center in Spain, over a 5-year period (2021–2025). All episodes of biliary-source bloodstream infection (bBSI) occurring in patients with active oncologic disease were included. Active oncologic disease was defined as receipt of chemotherapy, radiotherapy, immunotherapy, or palliative treatment within 3 months preceding the BSI episode, or surgical management of the malignancy within the previous 6 months.

### 4.2. Data Collection and Definitions

Data were collected using a standardized electronic case report form within the REDCap^®^ platform (version 16.0.16). Variables included demographic characteristics, major comorbidities, oncologic disease details (tumor site and stage), clinical presentation, microbiological findings, antimicrobial therapy, and clinical outcomes.

Previous biliary tract manipulations referred to endoscopic retrograde cholangiopancreatography (ERCP) or percutaneous transhepatic cholangiography (PTC). Prior bBSI was defined as an episode occurring within the previous year. Prior hospitalization for suspected biliary infection within the preceding year was recorded based on clinical judgment, implying the absence of BSI during that admission. Septic shock was defined according to the Sepsis-3 criteria [16].

Microbiological data included causative organisms and their antimicrobial resistance profiles. MDR Gram-negative bacilli were defined as: (i) Enterobacterales producing AmpC β-lactamases, (ii) Enterobacterales producing extended-spectrum β-lactamases (ESBLs), (iii) Enterobacterales producing carbapenemases, or (iv) Non-fermentative GNB (i.e., *P. aeruginosa*, *Acinetobacter* spp., or *Stenotrophomonas maltophilia*) resistant to at least three antibiotic classes. Inappropriate empirical antibiotic treatment (IEAT) was defined as the absence of at least one antibiotic with in vitro activity against the isolated pathogen in the initial antibiotic regimen (without considering timing of administration, dosing adequacy, or source control). Secondary liver abscesses were considered related to the bBSI if diagnosed within the subsequent weeks. Recurrent BSI was defined as a new episode of biliary-source bacteremia occurring after initial clinical improvement, caused by a different pathogen or by the same pathogen when occurring at least 14 days after the first infection. Delays or interruptions in cancer treatment were assessed retrospectively in collaboration with treating oncologists. Mortality was defined as death from any cause within 30 days of bBSI onset.

### 4.3. Microbiological Methods

BSI episodes were defined according to standard institutional microbiological protocols, based on the isolation of a clinically relevant pathogen from blood cultures in the appropriate clinical context. Blood cultures were processed using the BACTEC FX systems (Becton-Dickinson, Franklin Lakes, NJ, USA) with incubation up to 5 days. Bacterial identification was performed by MALDI-TOF mass spectrometry (Bruker, Billerica, MA, USA). Antimicrobial susceptibility testing was carried out using either the Vitek-2 Compact automated microdilution system (bioMérieux, Marcy-l’Étoile, France) or the Etest method (AB Biodisk/bioMérieux, Solna, Sweden). ESBL production was suspected based on MIC results and confirmed by the double-disc synergy test [17] or NG-Test^®^ CTX-M Multi (NG-BioTech, Guipry-Messac, France). Carbapenemase production was detected phenotypically using the modified carbapenem inactivation method (mCIM) [18], combined with the NG-Test^®^ CARBA 5 lateral flow immunoassay (NG-BioTech), which identifies the five most common carbapenemases: KPC, OXA-48-like, VIM, IMP, and NDM [19]. Susceptibility and resistance interpretations followed current EUCAST breakpoints.

### 4.4. Statistical Analysis

Categorical variables were presented as counts and percentages, whereas continuous variables were expressed as means ± standard deviations (SDs) or medians with interquartile ranges (IQRs), as appropriate. Because patients could experience more than one bBSI, analyses of microbiological characteristics and 30-day mortality were conducted at the episode level (*n* = 199 episodes), whereas recurrence analyses were performed at the patient level (*n* = 136 patients), considering the first episode for each patient as the index episode. Group comparisons were conducted using the Pearson Chi-square test for categorical variables and either Student’s *t*-test or the Mann–Whitney U test for continuous variables.

Independent risk factors for bBSI recurrence and 30-day mortality were identified using separate multivariable logistic regression models. Variable selection was guided by clinical relevance and supported by univariable analysis (*p* < 0.05). A stepwise forward selection method was applied, and final models were restricted to a limited number of predictors to ensure parsimony and maintain an adequate events-per-variable ratio. Model calibration was assessed using the Hosmer–Lemeshow goodness-of-fit test, and discriminative ability was evaluated by calculating the area under the receiver operating characteristic (ROC) curve. Multicollinearity among covariates was assessed using variance inflation factors and tolerance statistics. Internal validation was performed using bootstrap resampling (1000 samples, bias-corrected and accelerated confidence intervals). A two-tailed *p*-value < 0.05 was considered statistically significant.

To externally validate the previously published risk score for recurrent bBSIs, we assessed its predictive performance in our oncologic cohort. Following the original scoring system derived from multivariable regression coefficients, 1 point was assigned for the presence of a biliary prosthesis and for bBSI caused by an MDR-GNB, while 2 points were assigned for prior antibiotic therapy (within the last month), and for hospital admission due to suspected biliary infection. The discriminatory power of the clinical prediction rule was evaluated using the area under the ROC curve, and different cutoff points were explored to determine their corresponding sensitivity, specificity, and predictive values.

All statistical analyses were performed using SPSS software (version 25.0; SPSS, Inc., Chicago, IL, USA).

## 5. Conclusions

bBSIs in cancer patients are associated with high mortality and morbidity, often leading to delays or discontinuation of oncologic treatment. These episodes are frequently caused by Gram-negative bacilli, with a high prevalence of multidrug-resistant organisms. Inappropriate empirical antibiotic therapy was a modifiable factor independently associated with mortality, underscoring the need for risk-adapted empirical treatment strategies. Recurrence was common and driven by biliary tract malignancy, prior biliary infection, and previous MDR isolation. A simple clinical score identifies patients at high risk of recurrence and may support targeted preventive strategies in this high-risk population.

## Figures and Tables

**Table 1:** Cohort characteristics, oncologic features, and predisposing factors.

	Total Episodes N = 199 (%)	Non-Recurrent Episodes N = 88 (%)	Recurrent Episodes N = 48 (%)	*p*-Value	Alive N = 150 (%)	Dead N = 49 (%)	*p*-Value
Age (median, IQR ^1^; years)	71 (IQR 64–76)	64 (72.7%) ^1^	34 (70.8%) ^1^	0.814	104 (69.3)	39 (79.6)	0.166
Male sex	121 (60.8)	58 (65.9)	28 (58.3)	0.381	92 (61.3)	29 (59.2)	0.789
Comorbidities Diabetes Heart disease Lung disease Renal failure Stroke Liver disease	91 (45.7) 36 (18.1) 27 (13.6) 19 (9.5) 14 (7) 10 (5)	29 (33.0) 17 (19.3%) 15 (17.0) 10 (11.4) 5 (5.7) 3 (3.4)	24 (50.0) 10 (20.8) 6 (12.5) 3 (6.3) 6 (12.5) 5 (10.4)	0.51 0.832 0.483 0.543 0.195 0.130	72 (48.0) 29 (19.3) 16 (10.7) 14 (9.3) 9 (6.0) 5 (3.3)	19 (45.7) 7 (14.3) 11 (22.4) 5 (10.2) 5 (10.2) 5 (10.2)	0.260 0.426 0.037 0.787 0.339 0.069
Solid neoplasm Pancreatic Biliary Colorectal Gastro-esophageal Hepatic Other	73 (36.7) 66 (33.2) 21 (10.6) 8 (4.0) 6 (3.0) 25 (12.6)	34 (38.6) 22 (25.0) 4 (4.5) 4 (4.5) 5 (5.7) 19 (21.6)	17 (35.4) 20 (41.7) 7 (14.6) 1 (2.1) 1 (2.1) 2 (4.2)	0.711 0.440 0.520 0.656 0.424 0.006	56 (37.3) 44 (29.3) 19 (12.7) 6 (4.0) 5 (3.3) 20 (13.3)	17 (34.7) 22 (44.9) 2 (4.1) 2 (4.1) 1 (2.0) 5 (10.2)	0.739 0.045 0.111 1.000 1.000 0.804
Disease status Responding Stable Relapse/Refractory New diagnosis	15 (7.5) 27 (13.6) 92 (46.2) 65 (32.7)	2 (2.3) 18 (20.5) 32 (36.4) 36 (40.9)	5 (10.4) 5 (10.4) 28 (58.3) 10 (20.8)	0.096 0.158 0.014 0.018	14 (9.3) 26 (17.3) 66 (44.0) 44 (29.3)	1 (2.0) 1 (2.0) 26 (53.1) 21 (42.9)	0.123 0.004 0.323 0.080
Metastatic disease	121 (60.8)	47 (53.4)	33 (68.8)	0.082	84 (56.0)	37 (75.5)	0.015
Biliary tract involvement Primary Adenopathic Metastatic None	138 (69.3) 7 (3.5) 36 (18.1) 18 (9.0)	59 (67.0) 2 (2.3) 12 (13.6) 15 (17.0)	34 (70.8) 3 (6.3) 10 (20.8) 1 (2.1)	0.650 0.345 0.276 0.010	100 (66.7) 7 (4.7) 26 (17.3) 17 (11.3)	38 (77.6) 0 (0.0) 10 (20.4) 1 (2.0)	0.151 0.197 0.627 0.080
Oncologic treatment Chemotherapy Immunotherapy Hormonotherapy None	76 (38.2) 11 (5.5) 4 (2.0) 106 (53.3)	29 (33.0) 6 (6.8) 4 (4.5) 48 (54.5)	20 (41.7) 2 (4.2) 0 (0) 26 (54.2)	0.312 0.712 0.297 0.966	64 (42.7) 10 (6.7) 4 (2.7) 70 (46.7)	12 (24.5) 1 (2.0) 0 (0.0) 36 (73.5)	0.023 0.299 0.574 0.001
Community BSI Healthcare BSI Nosocomial BSI	52 (26.1) 67 (33.7) 80 (40.2)	29 (33.0) 31 (35.2) 28 (31.8)	8 (16.7) 16 (33.3) 24 (50)	0.041 0.824 0.037	43 (28.7) 51 (34.0) 56 (37.3)	9 (18.4) 16 (32.7) 24 (49.0)	0.154 0.862 0.149
Predisposing factors Previous admission ^2^ Previous surgery ^2^ Previous ICU admission ^2^ Neutropenia Corticosteroids Biliary manipulation ^2^ Biliary prosthesis Prior antibiotic ^2^ Antibiotic at BSI onset	102 (51.3) 26 (13.1) 11 (5.5) 2 (1.0) 8 (4.0) 121 (60.8) 128 (64.3) 83 (41.7) 47 (23.6)	32 (36.4) 11 (12.5) 4 (4.5) 2 (2.3) 1 (1.13) 46 (52.3) 49 (55.7) 21 (23.9) 11 (12.5)	33 (68.7) 6 (12.5) 3 (6.25) 0 (0.0) 6 (12.5) 35 (72.9) 37 (77.1) 30 (62.5) 18 (37.5)	<0.001 1.000 0.701 - 0.007 0.019 0.016 <0.001 <0.001	75 (50.0) 23 (15.3) 9 (6.0) 2 (1.3) 5 (3.4) 92 (61.3) 95 (63.3) 62 (41.3) 33 (22.0)	27 (55.1) 3 (6.1) 2 (4.2) 0 (0.0) 3 (6.1) 29 (59.2) 33 (67.3) 21 (42.9) 14 (28.6)	0.535 0.141 1.000 1.000 0.411 0.789 0.611 0.851 0.347

^1^: Age > 65 years; ^2^: During the last month.

**Table 2:** Microbiological results according to recurrence and 30-day mortality.

	Total Episodes N = 199 (%)	Non-Recurrent Episodes N = 88 (%)	Recurrent Episodes N = 48 (%)	*p*-Value	Alive N = 150 (%)	Dead N = 49 (%)	*p*-Value
Prior MDR isolates ^1^	20 (10.1)	3 (3.4)	15 (31.3)	<0.001	14 (9.3)	6 (12.2)	0.566
Gram negative bacilli	160 (80.4)	71 (80.7)	39 (81.2)	0.936	121 (80.7)	39 (79.6)	0.869
*E. coli* ESBL Carbapenem-R ^2^	86 (43.2) 19 (22.1) ^3^ 2 (2.3) ^3^	36 (40.9) 6 (6.8) 0 (0.0)	22 (45.8) 6 (12.5) 1 (2.1)	0.579 0.333 0.368	65 (43.3) 14 (9.3) 0 (0.0)	21 (42.9) 5 (10.2) 2 (4.1)	0.953 1.000 0.055
*K. pneumoniae* ESBL Carbapenem-R	48 (24.1) 12 (25.0) ^3^ 1 (2.1) ^3^	22 (25.0) 5 (5.7) 0 (0.0)	9 (18.8) 2 (4.2) 0 (0.0)	0.406 1.000 -	37 (24.7) 9 (6.0) 1 (0.7)	11 (22.4) 3 (6.1) 0 (0.0)	0.753 1.000 1.000
*Enterobacter* spp. ESBL	16 (8.0) 3 (18.8) ^3^	9 (10.2) 3 (3.4)	3 (6.3) 0 (0.0)	0.539 0.509	10 (6.7) 1 (0.7)	6 (12.2) 2 (4.1)	0.231 0.518
Other *Klebsiella* spp.	9 (4.5)	4 (4.5)	1 (2.1)	0.656	8 (5.3)	1 (2.0)	0.457
*P. aeruginosa* MDR	7 (3.5) 1 (14.3) ^3^	5 (5.7) 0 (0.0)	2 (4.2) 1 (2.1)	1.000 0.286	4 (2.7) 0 (0.0)	3 (6.1) 1 (2.0)	0.366 0.429
*Citrobacter* spp.	5 (2.5)	4 (4.5)	0 (0.0)	0.297	4 (2.7)	1 (2.0)	1.000
Other GNB ^4^	12 (6.0)	3 (3.4)	4 (8.3)	0.243	10 (6.7)	2 (4.1)	0.734
Gram positive cocci	56 (28.1)	25 (28.4)	15 (31.25)	0.728	36 (24.0)	20 (40.1)	0.023
*E. faecium*	38 (19.1)	13 (14.8)	11 (22.9)	0.234	25 (51.0)	13 (26.5)	0.127
*Streptococcus* spp.	11 (5.5)	8 (9.1)	2 (4.2)	0.494	6 (4.0)	5 (10.2)	0.143
*E. faecalis*	9 (4.5)	6 (6.8)	2 (4.2)	0.712	6 (4.0)	3 (6.1)	0.692
*Candida* spp.	3 (1.5)	-	-	-	3 (2.0)	0 (0.0)	1.000
Polymicrobial	41 (20.6)	21 (23.9)	7 (14.6)	0.201	27 (18.0)	14 (28.6)	0.112
MDR-GNB	38 (19.1)	15 (17.0)	10 (20.8)	0.586	25 (16.7)	13 (26.5)	0.127

^1^ During the previous 6 months, 20 patients had at least one MDR isolate, including ESBL *E. coli* (n = 10), ESBL *K. pneumoniae* (n = 10), carbapenemase-producing *E. coli* (n = 1), other ESBL Enterobacterales (n = 1), other carbapenemase-producing Enterobacterales (n = 2), and other MDR organisms (n = 2). Some patients had more than one isolate. ^2^ Carbapenem-resistant. ^3^ Percentage calculated over the total number of isolates of the species. ^4^ Including *Bacteroides* spp. (n = 3), *Aeromonas* spp. (n = 3), *Serratia* spp. (n = 2), *Morganella* spp. (n = 1), *Acinetobacter baumannii* (n = 1), *Stenotrophomonas maltophilia* (n = 1), and *Fusobacterium* spp. (n = 1).

**Table 3:** Main outcomes regarding recurrence and 30-day mortality.

	Total Episodes N = 199 (%)	Non-Recurrent Episodes N = 88 (%)	Recurrent Episodes N = 48 (%)	*p*-Value	Alive N = 150 (%)	Dead N = 49 (%)	*p*-Value
IEAT ^1^	74 (37.2)	32 (36.4)	16 (33.3)	0.724	49 (32.7)	25 (51.0)	0.021
Prior biliary BSI ^2^	71 (35.7)	-	-	-	52 (34.7)	19 (38.8)	0.602
Same bacteria causing prior biliary BSI	34 (47.9) ^3^	-	-	-	23 (15.3)	11 (22.4)	0.308
Prior biliary infection (without BSI) ^5^	83 (41.7)	15 (17.0)	39 (81.3)	<0.001	64 (42.7)	19 (38.8)	0.632
Biliary drainage requirement	80 (40.2)	31 (35.2)	18 (37.5)	0.792	67 (44.7)	13 (26.5)	0.025
Secondary hepatic abscess	38 (19.1)	16 (18.2)	10 (20.8)	0.707	30 (20.0)	8 (16.3)	0.570
Septic shock	38 (19.1)	26 (29.5)	7 (14.6)	0.052	22 (14.7)	16 (32.7)	0.005
ICU admission	14 (7.0)	9 (10.2)	4 (8.3)	1.000	10 (6.7)	4 (8.2)	0.750
Oncological treatment delay	23 (25.3) ^6^	9 (10.2)	4 (8.3)	0.364	23 (57.5)	0 (0.0)	0.439
Oncological treatment discontinuation	48 (52.7) ^6^	17 (19.3)	15 (31.3)	0.117	36 (24.0)	12 (24.5)	0.945
30-day mortality	49 (24.6)	27 (30.7)	18 (37.5)	0.419	-	-	-
bBSI-related mortality	37 (75.5) ^7^	20 (22.7)	13 (27.1)	1.000	-	-	-

^1^ IEAT: inappropriate empirical antibiotic therapy. ^2^ Prior biliary-source bacteremia. ^3^ Percentage among those episodes with prior biliary-source BSI. ^5^ Prior admission for suspected non-bacteremic biliary tract infection. ^6^ Percentage of all patients receiving active oncologic treatment. ^7^ Percentage of total deaths.

**Table 4:** Sensitivity, specificity, and predictive values of different operating cut-off points for predicting relapsing biliary-source bloodstream infection.

Score	*n*	Sensitivity	Specificity	PPV	NPV
>0	103	93.8%	34.1%	43.7%	90.9%
>1	72	89.6%	67.0%	59.7%	92.2%
>2	61	83.3%	76.1%	65.6%	89.3%
>3	44	62.5%	84.1%	68.2%	80.4%
>4	34	50.0%	88.6%	70.6%	76.5%
>5	7	6.25%	95.45%	42.9%	65.1%

Abbreviations. PPV: positive predictive value; NPV: negative predictive value.

## Data Availability

The data supporting the findings of this study are available from the corresponding author upon reasonable request, due to ethical and privacy restrictions related to patient data anonymization.

## Supplementary Materials

The following supporting information can be downloaded at: https://www.mdpi.com/article/10.3390/antibiotics15040342/s1, Table S1: Most frequently prescribed empirical antibiotic regimens; Table S2. Most frequently prescribed definitive antibiotic regimens.

## Abbreviations

The following abbreviations are used in this manuscript:

AEAT	Appropriate empirical antibiotic treatment
bBSI	Biliary-source bloodstream infection
BSI	Bloodstream infection
ESBL	Extended-spectrum β-lactamase
GNB	Gram-negative bacilli
ICU	Intensive care unit
IEAT	Inappropriate empirical antibiotic therapy
IQR	Interquartile range
MDR	Multidrug-resistant
MDR-GNB	Multidrug-resistant Gram-negative bacilli
NPV	Negative predictive value
OR	Odds ratio
PPV	Positive predictive value
