# Supplementary material for: Retrospective Analysis of the Epidemiology and Risk Factors for Recurrent Biliary-Source Bloodstream Infections in Oncologic Patients
Source: Antibiotics (Basel). 2026 Mar 27;15(4):342. doi: 10.3390/antibiotics15040342 (PMC13114129; doi:10.3390/antibiotics15040342)
Supplement: Supplementary file 1 [file antibiotics-15-00342-s001.zip › antibiotics-4170439-supplementary.pdf]

Supplementary table S1. Most frequently prescribed empirical antibiotic regimens.

Empirical antibiotic treatment	**Total episodes N=199 (%)**
Piperacillin/tazobactam	104 (52.3)
Meropenem	45 (22.6)
Vancomycin	19 (9.5)
Amoxicillin/clavulanate	13 (6.5)
Metronidazole	11 (5.5)
Amikacin	9 (4.5)
Ciprofloxacin	6 (3.0)
Linezolid	6 (3.0)
Ceftriaxone	5 (2.5)
Aztreonam	4 (2.0)
Cefepime	2 (1.0)
Ceftolozane/tazobactam	1 (0.5)
Ceftazidime/avibactam	1 (0.5)
Daptomycin	1 (0.5)
Other antibiotics	1 (0.5)
Anti–*E. faecium* coverage[a]	26 (13.1)
No empirical antibiotic therapy	19 (9.5)

[a] Including vancomycin, linezolid, and daptomycin.

Supplementary table S2. Most frequently prescribed definitive antibiotic regimens.

Definitive antibiotic treatment	Total episodes N=199 (%)
Ciprofloxacin	41 (20.6)
Amoxicillin/clavulanate	39 (19.6)
Meropenem	36 (18.1)
Piperacillin/tazobactam	30 (15.1)
Vancomycin	29 (14.6)
Metronidazole	25 (12.6)
Ceftriaxone	17 (8.5)
Linezolid	12 (6.0)
Ertapenem	8 (4.0)
Cefepime	4 (2.0)
Amikacin	4 (2.0)
Daptomycin	3 (1.5)
Aztreonam	2 (1.0)
Ampicillin	1 (0.5)
Ceftazidime/avibactam	1 (0.5)
Tigecycline	1 (0.5)
Levofloxacin	1 (0.5)
Other antibiotics	15 (7.5)
